# Simultaneous Integrated Boost (SIB) vs. Sequential Boost in Head and Neck Cancer (HNC) Radiotherapy: A Radiomics-Based Decision Proof of Concept

**DOI:** 10.3390/jcm12062413

**Published:** 2023-03-21

**Authors:** Camil Ciprian Mireștean, Roxana Irina Iancu, Dragoș Petru Teodor Iancu

**Affiliations:** 1 Department of Oncology and Radiotherapy, University of Medicine and Pharmacy Craiova, 200349 Craiova, Romania; 2 Department of Surgery, Railways Clinical Hospital Iasi, 700506 Iași, Romania; 3 Oral Pathology Department, Faculty of Dental Medicine, “Gr. T. Popa” University of Medicine and Pharmacy, 700115 Iași, Romania; 4 Department of Clinical Laboratory, “St. Spiridon” Emergency Universitary Hospital, 700111 Iași, Romania; 5 Oncology and Radiotherapy Department, Faculty of Medicine, “Gr. T. Popa” University of Medicine and Pharmacy, 700115 Iași, Romania; 6 Department of Radiation Oncology, Regional Institute of Oncology, 700483 Iași, Romania

**Keywords:** radiomics, artificial intelligence, radiotherapy, radio-chemotherapy head and neck cancers, SIB, VMAT, IMRT

## Abstract

Artificial intelligence (AI) and in particular radiomics has opened new horizons by extracting data from medical imaging that could be used not only to improve diagnostic accuracy, but also to be included in predictive models contributing to treatment stratification of cancer. Head and neck cancers (HNC) are associated with higher recurrence rates, especially in advanced stages of disease. It is considered that approximately 50% of cases will evolve with loco-regional recurrence, even if they will benefit from a current standard treatment consisting of definitive chemo-radiotherapy. Radiotherapy, the cornerstone treatment in locally advanced HNC, could be delivered either by the simultaneous integrated boost (SIB) technique or by the sequential boost technique, the decision often being a subjective one. The principles of radiobiology could be the basis of an optimal decision between the two methods of radiation dose delivery, but the heterogeneity of HNC radio-sensitivity makes this approach difficult. Radiomics has demonstrated the ability to non-invasively predict radio-sensitivity and the risk of relapse in HNC. Tumor heterogeneity evaluated with radiomics, the inclusion of coarseness, entropy and other first order features extracted from gross tumor volume (GTV) in multivariate models could identify pre-treatment cases that will benefit from one of the approaches (SIB or sequential boost radio-chemotherapy) considered the current standard of care for locally advanced HNC. Computer tomography (CT) simulation and daily cone beam CT (CBCT) could be chosen as imaging source for radiomic analysis.

## 1. Introduction

The simultaneous integrated boost (SIB) and the sequential boost technique are dose delivery strategies both for step and shot intensity-modulated radiation therapy (IMRT) and for volumetric modulated arc therapy (VMAT). Both strategies are used in daily clinical practice and the criteria for choosing one or other method are generally subjective, without clear recommendations for patient selection, although the biological effects of irradiation may be favorable for one technique or another, determined case by case. Head and neck cancers (HNC) are associated with higher recurrence rates, especially in advanced stages. It is considered that approximately 50% of cases will evolve with loco-regional recurrence even if they benefit from a standard treatment consisting of definitive chemo-radiotherapy. Although the subtype of HNC associated with human papilloma virus (HPV) infection has a more favorable prognosis and response to treatment compared to HNC associated with a long history of smoking, the “in field” recurrence rate remains higher for both subtypes. A total of 45% of loco-regional failures is identified in areas that received a median radiation dose of 67 Gy. The identification of parameters that could contribute to improving the results of multimodal treatment by increasing the rates of tumor control and also by reducing the treatment related toxicities in currently a topic of interest in HNC research. Radiomics, a method that uses artificial intelligence (AI) algorithms to extract data from high-resolution medical imaging with potential of diagnostic, predictive and prognostic value could also be implemented for modulating the treatment of HNC. We propose to argue the possibility of using radiomics for choosing the “sequential boost” technique or SIB IMRT/VMAT as the optimal technique for HNC radiotherapy [1,2,3,4].

## 2. SIB vs. Sequential Boost IMRT/VMAT in HNC—From “In Silico” Treatment Planning to Clinical Results

The sequential boost technique consists in the delivery of a single 2 Gy fraction per day, 5 days a week, 7 weeks typically, treating different target volumes with different dose levels. The “shrinking-field” procedure involves the initial irradiation of the entire planned volume including the elective one followed by the subsequent irradiation of only higher risk of recurrence target volumes. The SIB technique involves the irradiation of all target volumes during the entire treatment period, different dose levels being delivered simultaneously in different target volumes. Radiotherapy planning is based on CT simulation and delineation of the gross tumor volume (GTV) using the acquired images as support, but also adding clinical and imaging information supplemented and completed by other diagnostic methods. Macroscopically involved lymph nodes are recommended to be included in the GTV. The delineation of the clinical target volume (CTV) involves the rationale of including the specific risk regions of the microscopic disease invasion, taking into account the anatomical barriers of tumor invasion. It is generally accepted that an expansion of 1–1.5 cm is created around the GTV in order to obtain the CTV. The guidelines of the Radiation Therapy Oncology Group (RTOG) provide a consensus for the delineation of lymph node levels considered with higher and respectively lower risk of tumor involvement. Most often, lymph node level II-IV bilaterally are included in higher risk target volume. Typically, regionally node-negative target volumes are irradiated prophylactically with different dose levels depending on the risk of tumor metastases [5,6].

The standard fractionation regimen includes dose levels of 66–70 Gy to gross disease and doses of 45–60 Gy to non-involved lymph nodes, a fractionation regimen derived from older 3D-conformal (3D-CRT) technique being a feasible alternative. A standard regime consists in a three-phase sequence within total dose of 70 Gy in 35 daily fractions delivered on the target volume of gross disease, a total dose of 60–66 Gy in 30–33 daily fractions delivered to lymph nodes with a high risk of tumor invasion and a 50–56 Gy in 25–28 daily fractions for lower risk target volumes. Initially, the SIB technique was tested in clinical practice more than 15 years ago and was based on the RTOG protocol H-0022. The proposed fractionation schemes used fractional doses of 2 Gy, 2.2 Gy and 2.11 Gy up to 60–70 Gy, 66–68.2 Gy and, respectively, 69 Gy. Delivering a lower radiation dose in the same number of fractions to the elective target volume, the dose per fraction could decrease to 1.6–1.8 Gy per fraction. Recently, the results of NRG/RTOG 0022, the first trial that evaluated the possible benefits of IMRT in a multicenter study, highlighted high survival rates at 10 years (2/3 of the cases), the rate of grade 3–4 toxicities being reduced (from 1 to 7%). Overall survival (OS), disease-free survival (DFS) and local-regional failure (LRF) ratio was 67%, 50% and 15%, respectively. It should be mentioned that at the time of initiation of the study, the IMRT technique was mainly focused on optimizing the dose to the parotid glands. It is estimated that by identifying other critical structures and due to the implementation of some radiobiological models that also involve other toxicities with the exception of xerostomia, the treatment results will bring long-term benefits in relation to tardive toxicity [7]. More than 20 years ago, Mohan et al. demonstrated superior dose coverage of target volumes in SIB-IMRT plans for HNC. It should also be mentioned that the superior conformity in SIB-IMRT is also justified by the fact that the sequential IMRT technique involves the generation of a second plan for the boost volume, this plan having a dose distribution independent of the initial plan that includes the irradiation of elective volumes. Taking into account the lag time before the onset of accelerated tumor repopulation evaluated between 9 and 30 days, the SIB technique does not negatively influence the outcome of the treatment, its duration being considered sufficient to not require an addition of 0.5–0.7 Gy compensatory dose for each day of accelerated tumor repopulation. Considering values of at least 15 Gy for α/β for the tumor, the dose variation per fraction between 1.6 Gy and 3.0 Gy is not an important factor influencing the treatment result. However, at that time, the authors expressed their concern for the late effects for bone and muscle considering α/β values 0.85 and 3.1. The high values of α/β for the mucosa do not justify a significant influence of the dose per fraction, but the overall treatment time can be a factor that affects acute and late mucosal toxicity as a consistent effect [8]. Pharyngeal constrictor muscles, supra-glottic larynx and glottic larynx were identified as structures involved in dysphagia in a study that included 26 patients treated with radiotherapy concurrent with gemcitabine. The functioning of these anatomical structures was evaluated using swallowing with video-fluoroscopy, direct endoscopy and computer tomography (CT). Comparing the irradiation techniques, the standard IMRT technique offered a moderate protection of the structures involved in dysphagia in relation to the 3D-conformal (3D-CRT) technique, a superior result being obtained if the IMRT plans are optimized to reduce dysphagia. In the case of pharyngeal constrictors, the IMRT technique reduces the volume, receiving at least 50 Gy (V50) by 10%, and the optimization adds another 10% reduction in V50 compared to the 3D-CRT technique. In the case of the supra-glottic and glottic larynx, the optimization of IMRT plans generated an average benefit of 18% compared to the 3D-CRT technique in reducing the V50 value. Comparing the irradiation techniques, the standard IMRT technique offered a moderate protection of the structures involved in dysphagia in relation to the 3D-CRT technique, a superior result being obtained if the IMRT plans are optimized in order to reduce dysphagia. In the case of pharyngeal constrictors, the IMRT technique reduces by 10% and the optimization adds an additional 10% reduction in V50 compared to the 3D-CRT technique. In the case of the larynx (supra-glottic and glottic), the optimization of IMRT plans brought an average benefit of 18% compared to the 3D-CRT technique [9]. Ever since 2006, an early period of SIB-IMRT in HNC, Studer et al. reported, in a study that includes 115 cases, tumor control rates similar to the historical ones and a favorable toxicity profile in relation to the 3D-CRT technique [10]. The study could not perform an objective comparison between SIB and sequential boost IMRT from the point of view of toxicities and especially of parotid gland sparing, an already recognized advantage of the sequential IMRT technique at that moment. The authors recommended caution when a 2.2 Gy per fraction is used to irradiate larger volumes involving laryngeal structures. The study by Wu and collaborators had already demonstrated the ability of the SIB-IMRT technique to obtain a satisfying dosimetric coverage of target volumes and the possibility of avoiding at least one of the parotid glands [11]. Four dosimetric parameters were assessed: biologically equivalent uniform dose (EUD), dose to specified percent (X) of volumes (Dx), homogeneity index, defined as the ratio D2–D98/prescription dose, and PITV, a compliance index defined as the volume covered by the prescribed isodose/target volume. The SIB technique also demonstrated the ability to deliver the radiation dose with increased homogeneity in the boost target volume, but delivered a relatively heterogeneous radiation dose in the elective treated volume. SIB is also considered feasible by the ability to limit the dose to the spinal cord to <38 Gy in the equivalent dose to 2 Gy standard regimen. The parotid glands could be spared, but the ability of the SIB to reduce the dose received by them depends on their degree of inclusion of parotid glands in the planning target volume (PTV). For not included or partially included parotid glands in PTV, SIB-IMRT could limit the dose to 2/3 of the volume to <32 Gy using dose conversion in the standard 2 Gy regimen by linear quadratic (LQ) radiobiologic model. Lauve et al. reported an average mean dose of 32Gy for the parotid gland in the vicinity of PTV and an average mean dose of 24 Gy for the contralateral parotid gland [12,13]. A dose regimen of 70.8 Gy in 30 fractions of 2.36 Gy delivered on the gross disease was associated with primary tumor, nodal and distant control rates of 76.3%, 66.7%, and 71.8%, respectively. With a 2% rate of late grade 3–4 toxicity, SIB-VMAT is considered a feasible technique for HNC cases aged 80+ [14,15].

A retrospective cohort analysis including patients with locally advanced HNC treated with definitive chemo-radiotherapy with doses of 69.3 Gy in 33 fractions compared sequential boost technique and SIB IMRT. The study that included 68 cases in the group treated with sequential boost regimen and 141 cases treated with SIB IMRT and identified a 4-year survival benefit in favor of SIB (76.8% vs. 69%). Even if gastrostoma dependence and weight loss were similar in the two groups of patients, the rates of dysphagia and dermatitis were 27% and 22%, respectively, higher in the SIB group. The relatively higher rate (7% vs. 0%) of treatment interruption due to acute toxicities in the SIB group should also be mentioned [5,16].

VMAT technique offers advantages in limiting the dose delivery time in the target volumes with a possible radiobiological benefit, which is a preferred solution in radiotherapy centers with longer waiting lists, but due to the scattering of small doses of radiation in large volumes of healthy tissue, it implies a possible additional risk of second malignancy. VMAT technique, based on the IMRT principle, could also offer dose delivery in both sequential boost and SIB mode. However, the data comparing SIB VMAT and VMAT with the sequential technique are relatively limited [17]. In a prospective interventional study that included 52 patients from India, authors evaluated comparative radio-chemotherapy (Cisplatin 40 mg/m^2^ plus VMAT radiotherapy) using the sequential boost technique and the SIB technique. Having similar inclusion criteria in the two trial arms, the dosimetric data were also similar. Acute toxicity rates (dermatitis, mucositis and dysphagia) were higher in the SIB-VMAT group with similar rates of local control (65.4% in the SIB group vs. 53.85% in the sequential boost VMAT group). The authors highlighted the comparable results between the two groups, also mentioning a possible technical advantage of the sequential boost VMAT regarding the treatment replanning facility. Assessing normal tissue complication probabilities (NTCP) for late dysphagia, with a radiobiological model proposed by Christianen et al., Cilla and collaborators evaluated the risk of dysphagia associated with SIB-VMAT treatment plans using total doses of 70.5 (67.5), 60.0 and 55.5 Gy delivered in 30 fractions. Considering the pharyngeal constrictor muscles (PCM) and glottic and supra-glottic larynx (SGL) as organs at risk (OARs), the SIB-VMAT plan focused on reducing dysphagia, providing a dose reduction of 3.9 Gy and 4.5 Gy to the superior pharyngeal constrictor muscles (uPCM), respectively SGL [17,18,19].

## 3. The Radiobiological Implications of SIB-IMRT/SIB-VMAT in HNC

Shortening the overall treatment time (OTT) and increasing the fraction size for the “boost” volume make the SIB technique a new approach with accelerated fractionation concept for the definitive multimodal treatment of HNC. Traditionally, accelerated fractionation is considered a reduction in OTT without significantly changing the dose per fraction, but usually the technique involves the administration of two fractions per day. A dose of 2.4 Gy per fraction is defined as the lower limit of moderate hypo-fractionation regimen. In some situations, for the target volume of gross disease, the SIB regimen combines the characteristics of these two concepts (the moderate hypo-fractionation and the accelerate fractionation). The concept of accelerated fractionation and reduction in treatment duration is based on lowering the risk of clonogenic regrowth in the last phase of treatment. From a radiobiological point of view, an accelerated irradiation regimen could bring benefits in term of tumor control for cases where the tumor clonogen’ survival time (Tpot) is <3 days. A maximum benefit is obtained if the OTT is shorter than the interval from the beginning of the treatment to the onset of accelerated repopulation. Values between 14 days and 3–5 weeks have been reported for this interval (T_K_). Tpot values for HNC are evaluated at 3–5 days, lower than the cutoff value of 5 days, above which the benefit of accelerated fractionation is considered minimal. A reduction in OTT below 30 days could be beneficial from the radiobiology point of view for the primary tumor. However, for acute responder tissue such as the mucosa, compensatory repopulation begins at the end of the first week of treatment and the risk of the early onset of grade 2–3 side effects is considered higher in the case of accelerated fractionation. Relatively higher α/β values (between 7 and 20), both for the tumor and for the acute responding tissue, make the dependence of the biological effects with the dose more linear, thus making the magnitude of toxicities less sensitive to the increase in doses per fraction. Values of 120–150 days until the start of accelerated repopulation for late responder tissues suggest that the late sequelae of accelerated fractionation on the mucosa and pharyngeal constrictor muscles is mainly attributed to a consequential effect, the correlation of some late effects with the severity of acute toxicities being already known [20,21,22,23,24].

Fractionation-dependent toxic effects are more severe for temporal lobe necrosis from nasopharyngeal carcinoma radiotherapy (α/β estimated at 2.9) and for laryngeal edema (α/β estimated at 2.35). Radiobiology studies also highlight a possible benefit of a high dose per fraction (2.5Gy) for early laryngeal cancer, tumoral type with an α/β estimated at 10. However, for doses >2.5 Gy per fraction, mucosal regeneration could be severely affected, the precautions in implementing SIB regimens that involve a dose of 2.4 Gy per fraction or higher in association with chemotherapy also being necessary [25,26]. However, the study by Schwartz et al., in which a dose of 60 Gy in 25 fractions on the target volume of the primary tumor was proposed, demonstrated superior rates of 83% of local control and 80% overall survival rates at 25 months with maximum rates of 55% of acute skin grade 2–3 toxicity for SIB-IMRT with or without concurrent chemotherapy. Higher local control rate 87% vs. 80% was obtained with a regimen of 67.2 Gy in 28 daily fractions for primary tumor plus involved nodes and 56 Gy in daily 28 fractions for elective irradiation vs. the SIB regimen of 63 Gy in 28 daily fractions for gross disease and 51.8 Gy in 28 daily fractions for elective irradiation [27].

Head and neck squamous cell carcinomas (HNSCC) associated with human papillomavirus (HPV), a subtype of HNC that is increasing in incidence, seems to be much more radiosensitive than HNC associated with smoking. Favorable response to definitive radio-chemotherapy encouraged the de-escalation trials, but until now, the negative results supported the use of standard treatment and not de-escalation for this subtype of HNC. The higher heterogeneity of HNC in terms of radio-sensitivity is highlighted by Reid and colleagues. Although some cell lines seem to be radiosensitive, repeated fractional irradiation seems to have the effect of increasing radio-resistance during treatment. The authors therefore consider that intrinsic radio-sensitivity should not be a decisive factor in the de-escalation of the treatment [28].

## 4. Radiomics and Artificial Intelligence (AI)—Perspectives in SIB or Sequential Boost Radiotherapy Decision in HNC

In the last decade, the computerized analysis of medical images has evolved exponentially, the conversion of images into data for decision-making purposes in medicine called radiomics. As mentioned by Gillies and his collaborators, “Images Are More than Pictures, They Are Data”, practically opening new horizons in the approach to medical imaging beyond visual analysis. The improvement of diagnostic power and the formulation of predictive and prognostic models make radiomics and cancer management a promising partnership [29].

The first step of the radiomic analysis is the segmentation of the image and the delineation of the region of interest (ROI) when using two-dimensional images and the volume of interest (VOI) for three-dimensional structures. The definition of ROIs/VOIs could be performed manually, semi-automatically or automatically. Manual and semi-automatic segmentations have the disadvantage of being time-consuming processes, but could also introduce sources of inter-observer errors. Automatic deep learning algorithms have already been integrated into applications for target volume delineation in radiotherapy, with most of the identified applications focused on head and neck organs at risk (OARs) and normal tissue structure segmentation. The second step of radiomics, the image processing, is an intermediate stage between the segmentation and the radiomic feature extraction. The purpose of this step is an attempt to standardize the images in terms of the distance between pixels, gray levels in order to increase the accuracy, and reproducibility of the radiomic algorithm. Interpolation to isotropic voxel spacing is an algorithm considered necessary to increase the reproducibility of extracted textural features, and range re-segmentation and intensity normalization are used to eliminate pixels or voxels that fall outside a gray range. Discretization of image intensities is necessary to group data in specific range intervals. The third stage, feature extraction and quantification of gray levels from the ROI/VOI, is recommended to be performed according to the recommendations of the Image Biomarker Standardization Initiative (IBSI) guidelines. The most frequently encountered radiomic features are based on intensity (histogram), shape and texture features. Wavelet or gaussian filters could also be used in this step. Feature selection/dimension reduction is the last step before creating the radiomic model. Reducing the number of non-reproducible, redundant and irrelevant features is an essential step in designing a performing radiomic model [30,31,32].

However, radiomics faces many challenges that limit its widespread application in clinical routine. The absence of standardization, the limited number of codes and open-source data, the lack of reporting and the difficulties in standardization are some of the causes of the difficulties in applying radiomics for diagnostic, predictive and prognostic purposes. Radiomics studies have successfully used all types of high-resolution medical images: computer tomography (CT), magnetic resonance imaging (MRI), positron emission tomography (PET), but also digital radiographs (XRD), ultrasonography or mammography. However, since most studies are retrospective, the uneven use of acquisition protocols and filters between institutions makes it difficult to report radiomics data. We should also mention “non-reducible technical variations” that involve patient variabilities that cause image noise or artifacts that are not dependent on the scanner settings and can affect the quality of the radiomics data [30,33,34].

The immense potential of radiomics in radiation oncology is presented in a systematic review by Bibault et al., who consider radiomics “a primer for radiation oncologist”. Using the search terms “radiotherapy”, “radiation oncology” and “radiomics” a search in the Medline database in 2019 identified in all the evaluated studies the previously mentioned steps of radiomics, with the fifth step considering the construction of the radiomic model. Most of the identified studies evaluated head and neck and lung cancers (five studies), followed by esophageal and rectal cancer (three studies), pancreatic cancer and brain metastases (two studies). The authors mention the heterogeneity of the data and that they have not been translated into clinical practice. The authors declared themselves optimistic about the future that will bring new robust and generalizable models [35]. Lohmann et al. mentioned the ability of radiomics to be implemented in neuroimaging with application in radiation oncology. Perfusion (PWI) and diffusion-weighted (DWI) MRI and PET functional images could bring an additional benefit for the model creation if associated with anatomical/structural imaging. The auto-contouring application based both on feature-based radiomics and deep learning are mentioned in particular, with three applications including open source PyRadiomics, MaZda, and LifeX for feature-based radiomics analysis also proposed for radiomic analyses [36,37,38,39].

In order to understand the perspectives of radiomics in radiation oncology for the construction of treatment response predictive models, we must mention that modern radiotherapy is based on the delivery of a homogeneous, tumoricidal dose in a well-defined region dose to surrounding normal tissue, an objective that can only be achieved with support of CT simulator, as mentioned by Iancu et al. almost 20 years ago [40]. Today, image-guided radiotherapy (IGRT) planning involves CT simulation and the on-board imaging (OBI) system attached to linear accelerators (LINAC) including both onboard kV and cone beam CT (CBCT) imaging devices as standard. A solid partnership between radiotherapy and radiomics is easy to anticipate involving the use of these imaging devices of the radiotherapy treatment planning/delivery chain [41,42].

A deep learning artificial neural networks (DL-ANN) model that analyzed pre-treatment CT images of HNC extracted from GTV and PTV aimed to predict cancer recurrence rate, but also death prognosis. The accuracy of the model built on the basis of pretreatment CT images from 188 HNC cases was estimated to be 77.7% and 74.3% if it was created based on features extracted from the PTV and GTV, respectively. The delivery of a personalized multimodal treatment, but also of radiotherapy according to the concept of precision medicine by enhancing efficacy and limiting toxicity, is one of the future perspectives identified by the authors. If the models use “classical” radiomics feature (features extracted and selected with a supervised algorithm) in favor of newer unsupervised deep learning concepts, entropy is reported as the most stable first-order features. However, for shape and textural features, there is no consensus regarding the choice of the most concordant and reproducible radiomic feature [43,44,45].

Radiomics was evaluated to differentiate the level of radio-sensitivity of different tumor sub-volumes in order to intensify the radiotherapy regimen in a study that included 40 cases. The fusion between PET/CT imaging for post-treatment evaluation with pre-treatment contrast agent-based CT images was used for the topographical localization of the tumor relapse regions. The patient lot was divided into the training set (28 cases) and the validation set (12 cases). The radiomic analysis aimed to compare the radiomic features extracted from uncontrolled GTV (which included data not correlated with control PET/CT) and data from sub-volumes correlated with recurrence PET/CT images. The radiomic features differed significantly between the two regions, but based on the radiomic features extracted from these regions, recurrence regions could also be anticipated. Radiomic analysis demonstrated the ability to identify regions of radio-resistance, and, consequently, CT radiomics can be a predictive biomarker in radiotherapy. Increased tumor heterogeneity was identified as a predictor of recurrence [46,47].

Non-rigid anatomical changes and the correction of the patient’s positioning during the treatment are guided with daily CBCT in IGRT. The identification of a radiomic signature in CBCT images was evaluated in a lot of 93 HNC cases, of which 60 cases were included in the training set, and the remaining 33 cases in the validation/test set. GTV was chosen as VOI and 88 radiomic features were analyzed in weekly dynamics. Initially, seven radiomic features were identified with an important dynamic during the radio-chemotherapy treatment. After excluding the inter-correlated features, only coarseness radiomic features was identified as a possible biomarker of treatment response. Coarseness is a measure of the difference between the center voxel and its neighborhood. Increased values of coarseness are associated with lower spatial change rate. Hemoglobin level was the predictive clinical variable for treatment response. The study advocates the use of multivariable models that include radiomic and clinical features for predicting the response to definitive radio-chemotherapy [47,48,49].

## 5. Model-Based Treatment Decision Framework: Are We Ready for AI-Based Model Implementation?

The purpose of the causal classification is to identify those cases that will associate a positive response in the case of a chosen treatment. Estimating the potential results includes a note of uncertainty by observing individuals under only one condition regarding the treatment, without being able to know exactly if certain cases were affected by that treatment. Fernández-Loría et al. mentioned the routine use of the simple prediction of the results in clinical practice and theoretically analyzed the possibility that the simple prediction is preferable to the estimation of the treatment effect. The result of the analysis underlined the necessity of the concept of causal variance compromise. The risk of error is considered proportional to the larger sampling variance if the result is dependent on two outcome predictions. In this case, but also in the situation that associate a stronger signal to noise ratio, a biased outcome prediction is preferred. The authors mentioned three scenarios in which the prediction of outcomes can be a valid option: the bias can be limited by changing the threshold, there is a correlation between the results and the effect of the treatment, and there are limited data for the evaluation of counterfactuals. All interventional models require both the correct specification of cause and effect, but also the identification of alternative scenarios called counterfactuals, conceptually being necessary to be applied in the case of models based on artificial intelligence. Prospri et al. mentioned two types of bias (confounding and collider). In the case of confounding, there is a variable that causes the effect and outcomes, inducing the wrong assumption that exposure to a factor caused the effect, even if in reality there is no cause–effect relationship [50,51,52].

The Dutch National Indication Protocol for Proton Therapy (NIPP) includes the evaluation of three NTCP models (≥grade 2 xerostomia, dysphagia and feed tubing dependence) and three NTCP thresholds, including NTCP delta; variations from 5 to 15% are used for the selection of patients who will benefit from fully reimbursed proton therapy. Although model-based selection is considered an accurate method to predict the benefit of significantly limiting treatment related toxicity by using proton therapy, it requires an in silico treatment planning and comparison that is both beneficial and cost-effective, but can also be associated with delaying the initiation of treatment. To prevent redundant protonotherapy planning, Tambas et al. proposed five methods based mainly on regression models, with the aim of predicting the mean dose (Dmean) for OARs in proton therapy plans, supporting VMAT plans. In conclusion, the study demonstrated that the advanced preselection tool based on VMAT plans can prevent laborious work in 68% of cases that would have been evaluated and planned for proton therapy and would not have qualified [52,53,54].

The concept of a semi-automatic NTCP Quality of Life (QoL)-weighted model based on head and neck cancers VMAT treatment plans optimization was proposed by van der Laan and colleagues in a study that included 30 cases. A total of 80 multivariable NTCP models including the evaluation of the 20 most frequent toxicities were evaluated at 6, 12, 18 and 24 months after the completion of radiotherapy. The plan optimization strategy was based on limiting the doses, especially to the organs that are involved in toxicities without affecting the dosimetric coverage of target volumes. Dysphagia, fatigue, speech problems, hoarseness and xerostomia were among the toxicities included in the semi-automatic plan optimization algorithm. The results of the study highlighted the greatest reduction in NTCP (−7.6% and −6.1%) with ≥grade 3 and ≥grade 2 dysphagia as the endpoint. However, the QoL-weighted optimization of VMAT plans resulted in an increase in NTCP related to xerostomia. The study predicted an improvement in QOL by 0.7, 0.9, 1 and 1.1 points using a scale from 1 to 100 at 6, 12, 18, 24 months, respectively [55].

Modern NTCP models include both a translation of the dose distribution associated with disease and patient characteristics into a probability of a complication occurring. The development and improvement of NTCP was associated with an increase in the interest for using these radiobiological models in clinical practice, these models being actively involved in the phase of treatment plan optimizing in order to guide the dose distribution. However, this approach requires an increased performance of the model and a concordance between the predicted probability of complications and the observed toxicity during the patient follow-up process. It is considered that both to obtain an increased probability when choosing an optimal technique, as well as to identify and select relevant predictors to be included in the model are essential objectives. NTCP modeling faces many challenges including missing data, non-linear responses, overfitting or predicting different degrees of toxicity at different time points. Missing data is a process that could be encountered both in the pre-treatment stage and in the post-radiotherapy follow-up. Multi-state models and multilevel imputation using joint models are examples of modern techniques used to compensate for this uncertainty factor. The inclusion of all baseline patients and tumor characteristics together with radiation dose values and their analysis in multivariate models, but also their evaluation through non-linear models, could increase the accuracy of the model. Classic models include predictors reported in the literature and in clinical data based on the idea of a linear relationship. Multi-collinearity involves the correlation of two or more variables included in the analysis and could be a disturbing factor in the selection of predictors [52,53].

Based on the dosimetric advantages of proton therapy which, due the generation of a Bragg peak, associates a flat dose profile in the vicinity of the target, Langendijk et al. proposed a randomized clinical trial (RCT) in order to evaluate whether the dose escalation with protons could bring a benefit without increasing the risk of treatment-related toxicity. A two-phase approach (a phase that includes patient selection, construction and evaluation of an NTCP model, in silico planning (ISPC) studies and benefit estimation and a second phase that includes clinical validation) is implemented in order to identify the plans that will be associated with a lower treatment-related toxicity, but with the same dose level for the purpose of tumor control. Even if an NTCP model-based approach cannot be considered routine to replace an RCT, a pre-treatment evaluation based on an NTCP model could have a higher level of prediction compared to the simple comparative evaluation of doses for two different treatment plans. However, it should be mentioned that there are confounding factors that are often not included in NTCP models (concurrent treatment with chemotherapy, two different dosimetric values that predict the same toxicity—as in the case of swallowing dysfunction) [7,52,53].

## 6. Conclusions

HNC is characterized by an increased heterogeneity as radio-sensitivity, and choosing an optimal treatment regimen is difficult in this context. Sequential boost or SIB IMRT/VMAT are both accepted regimens administered as standard treatment with comparable results, the choice of technique often being a subjective decision or based on dosimetric considerations. Radiomics has demonstrated the ability to non-invasively predict radio-sensitivity and the risk of recurrence in HNC. Tumor heterogeneity evaluated with radiomics analysis, the inclusion of coarseness, entropy and other first-order features extracted from GTV in multivariate models could identify pre-treatment cases that will benefit from one of the approaches (SIB or sequential boost radio-chemotherapy) considered the current standard of care for locally advanced HNC. CT simulation and daily CBCT could be chosen as imaging methods for radiomic analysis. Even if it does not offer the accuracy of RCT, the prediction of the outcome of a treatment based on NTCP models, already used in the selection of cases that will benefit significantly from proton beam therapy, but also the optimization of HNC plans focused on QoL, are already validated strategies and could be adopted in the case selection of SIB vs. sequential boost IMRT/VMAT for HNC. The inclusion of radiomic features in modern NTCP models will increase the accuracy of the prediction, important for the concept of precision medicine, even if currently both SIB and sequential are considered accepted variants in clinical practice.

## Data Availability

Not applicable.
